# Integration of horizontally acquired light-harvesting genes into an ancestral regulatory network in the cyanobacterium *Acaryochloris marina* MBIC11017

**DOI:** 10.1128/mbio.02423-24

**Published:** 2024-11-18

**Authors:** Nikea J. Ulrich, Scott R. Miller

**Affiliations:** 1 Division of Biological Sciences, University of Montana, Missoula, Montana, USA; Oregon State University, Corvallis, Oregon, USA

**Keywords:** cyanobacteria, novel trait, regulatory assimilation, Dollo's Law, conserved light response, *Acaryochloris marina*

## Abstract

The acquisition of new capabilities by horizontal gene transfer (HGT) shapes the distribution of traits during microbial diversification. In the Chlorophyll (Chl) *d*-producing cyanobacterium *Acaryochloris marina*, the genes involved in the production and disassembly of the light-harvesting phycobiliprotein phycocyanin (PC) were lost in the *A. marina* common ancestor but then subsequently regained via HGT in *A. marina* strain MBIC11017. However, it remains unknown how the HGT-acquired PC genes in MBIC11017 have been reintegrated into its existing regulatory network after tens of millions of years since their loss. Here, we investigated potential mechanisms of regulatory assimilation of PC genes by comparing the transcriptomes of *A. marina* strain MBIC11017 and a PC-lacking close relative under both low irradiance far-red light and high irradiance white light. We found that PC assembly and degradation processes have been re-assimilated into a conserved ancestral response to high light. Further, we identified putative regulatory elements that were likely co-transferred with PC genes and could be recognized by *A. marina*’s pre-existing light response machinery. This study offers insights into how HGT-acquired genes can be reintegrated into an existing transcriptional regulatory network that has evolved in their absence.

**IMPORTANCE:**

Horizontal gene transfer, the asymmetric movement of genetic information between donor and recipient organisms, is an important mechanism for acquiring new traits. In order for newly acquired gene content to be retained, it must be integrated into the genetic repertoire and regulatory networks of the recipient cell. In a strain of the Chlorophyll *d*-producing cyanobacterium *Acaryochloris marina*, the recent reacquisition of the genes required to produce the light-harvesting pigment phycocyanin offers a rare opportunity to understand the mechanisms underlying the regulatory assimilation of an acquired complex trait in bacteria. The significance in our research is in characterizing how an ancestrally lost, complex trait can be reintegrated into a conserved regulatory network, even after millions of years.

## INTRODUCTION

Horizontal gene transfer (HGT)—the asymmetric movement of genetic information between donor and recipient organisms, which in some cases may be only distantly related—is an important mechanism for acquiring new traits. In bacteria and archaea, genes can be horizontally acquired via direct cell-to-cell transfer of a plasmid (conjugation), taken up from the environment (transformation), or mediated by bacteriophage (transduction) [reviewed in reference (1)]. HGT has been pervasive throughout microbial evolution (2). This spans the ancient origins of eukaryotic organelles through endosymbiosis and subsequent genetic integration of entire organisms (3) to the modern spread of bacterial antibiotic resistance (4).

In order for newly acquired gene content to be retained, it must be integrated into the genetic repertoire and regulatory networks of the new host cell. This could involve the *de novo* rewiring of individual genes into a new network or, alternatively, co-option of gene clusters through the recruitment of an existing host network regulator (5, 6). Complex novel traits (i.e., traits whose development involves many interacting genes) generally involve the coordinated expression of many transcription factors and signaling pathways. Sometimes, HGT genes contain their neighbor *cis*-regulatory elements (e.g., promoters) that were co-transferred together. For example, in *Escherichia coli*, for cases in which HGT genes and their *cis*-regulatory elements were co-transferred, nearly half (17 out of 39 cases) of the operons were likely regulated by these co-inherited elements (7). However, HGT genes typically evolve new regulation, often from multiple regulators in their new host (7, 8). The majority of HGT-acquired genes in *E. coli* are regulated by two or more transcription factors (7), and often, upon acquisition, HGT genes rely on proteins such as histone-like nucleoid structuring proteins to be incorporated and embedded in the host’s regulatory networks (8). The functional integration of HGT genes into existing networks can span millions of years of evolution and often involves accelerated evolution of regulatory regions (9, 10); yet, our understanding of the integration process in the development of novel complex traits is still limited, particularly for non-model organisms.

Cyanobacteria are known for extensive HGT, especially with respect to light-harvesting genes (11–14). In the Chlorophyll (Chl) *d*-producing cyanobacterium *Acaryochloris marina*, we have shown that the genes involved in the production and degradation of the phycobiliprotein phycocyanin (PC) were lost in the *Acaryochloris* ancestor, but then subsequently regained via HGT by *A. marina* strain MBIC11017 (13). Complexes of bilin-containing phycobiliproteins (i.e., phycobilisomes; PBS) harvest photons that are not absorbed by Chl (15). Consequently, these genes allow MBIC11017 to grow in yellow and green light that is inaccessible to other *A. marina* (13). PC is composed of heterodimers of α and β peptides (encoded by *cpcAB*) that aggregate into hexamers (a trimer of heterodimers) and hexameric rods in association with linker proteins CpcC and CpcD (16). The PBS of MBIC11017 is a four-hexamer rod (17, 18) that efficiently transfers energy to photosystem II (PSII (19); and is anchored to the thylakoid membrane or the photosynthetic reaction center itself by CpcL via a C-terminal hydrophobic segment (20). MBIC11017 also contains Chl-binding proteins, the membrane-bound ancestral *A. marina* light-harvesting apparatus (13, 21). These Chl-binding proteins, homologous to the prochlorophyte Chl *a*/*b*-binding proteins (Pcb), serve as the major light-harvesting complexes in all other *A. marina*. It has been hypothesized that Pcbs and PC rods in MBIC11017 have competitive interactions with respect to pigment biosynthesis (22, 23). The acquisition and retention of PC genes in MBIC11017 suggest a selective advantage in the natural environment, and it remains the only isolated *A. marina* strain with this additional light-harvesting machinery (24).

This recent reacquisition by MBIC11017 of PC genes offers a rare opportunity to understand the mechanisms underlying the regulatory assimilation of an acquired complex trait in bacteria. PBS can contain up to half of the soluble cellular protein and nitrogen content of cyanobacteria (25, 26), and its assembly and degradation are tightly controlled by complex transcriptional changes in response to both light and nutrient levels (27–29). These regulatory genes are conserved in all *A. marina*, most likely due to their pleiotropic effects during cellular response to stress. Rapid degradation of PBS occurs under high light or nutrient limitation, particularly when triggered by nitrogen starvation (30). PBS degradation begins with the rapid transcriptional activation of *nblA*. NblA mediates the disassembly of PBS by interacting with phycobiliprotein subunits (31), and NblB detaches the bilin chromophores (32), such that the PBS proteins can be further disassembled by a Clp protease (33). NblS/RpaB, a highly conserved cyanobacterial two-component signal transduction system, controls transcriptional responses (including *nblA* expression) to various environmental signals (29, 34–36). The sensor histidine kinase NblS senses external stimuli and has been demonstrated to change the phosphorylation state of the response regulator RpaB (34, 37, 38). For example, dephosphorylation of RpaB in response to high light results in its release from the high light regulatory 1 (HLR1) sequence upstream of *nblA*, consequently de-repressing *nblA* transcription (39, 40). NblR is another conserved response regulator also involved in PBS degradation but has other functions related to survival in high light and during starvation (41). Hernandez-Prieto et al. (21) proposed that PC regulation in MBIC11017 is part of a general response to a change in light quality (white light [WL] vs far red [FR]) that signals planktonic growth and increased PC in WL and an attached lifestyle in FR. However, it is unknown how the HGT-acquired PC genes in MBIC11017 have been reintegrated into its existing transcriptional regulatory network following millions of years of *A. marina* diversification (42).

Here, we investigated whether PC regulation has been assimilated into an ancestral cyanobacterial high light to low light response. In high light, we would expect to observe a general downregulation of PC together with other photosynthesis genes, but higher expression of genes associated with light stress. We used a comparative transcriptomics approach under low irradiance FR (high PC) and high irradiance WL (low PC) between *A. marina* strain MBIC11017 and its PC-lacking close relative *A. marina* strain MU13 [strains are identical in sequence at the 16S rRNA gene, and their genomes differ at only ~1.7% of orthologous nucleotide positions (13)]. Ultimately, this study offers insights into how horizontally transferred genes are integrated into the transcriptional networks of recipient microorganisms to express novel complex traits that promote biological diversification.

## RESULTS AND DISCUSSION

### Differential expression between MBIC11017 and a close PC-lacking relative

**Fig 1:**
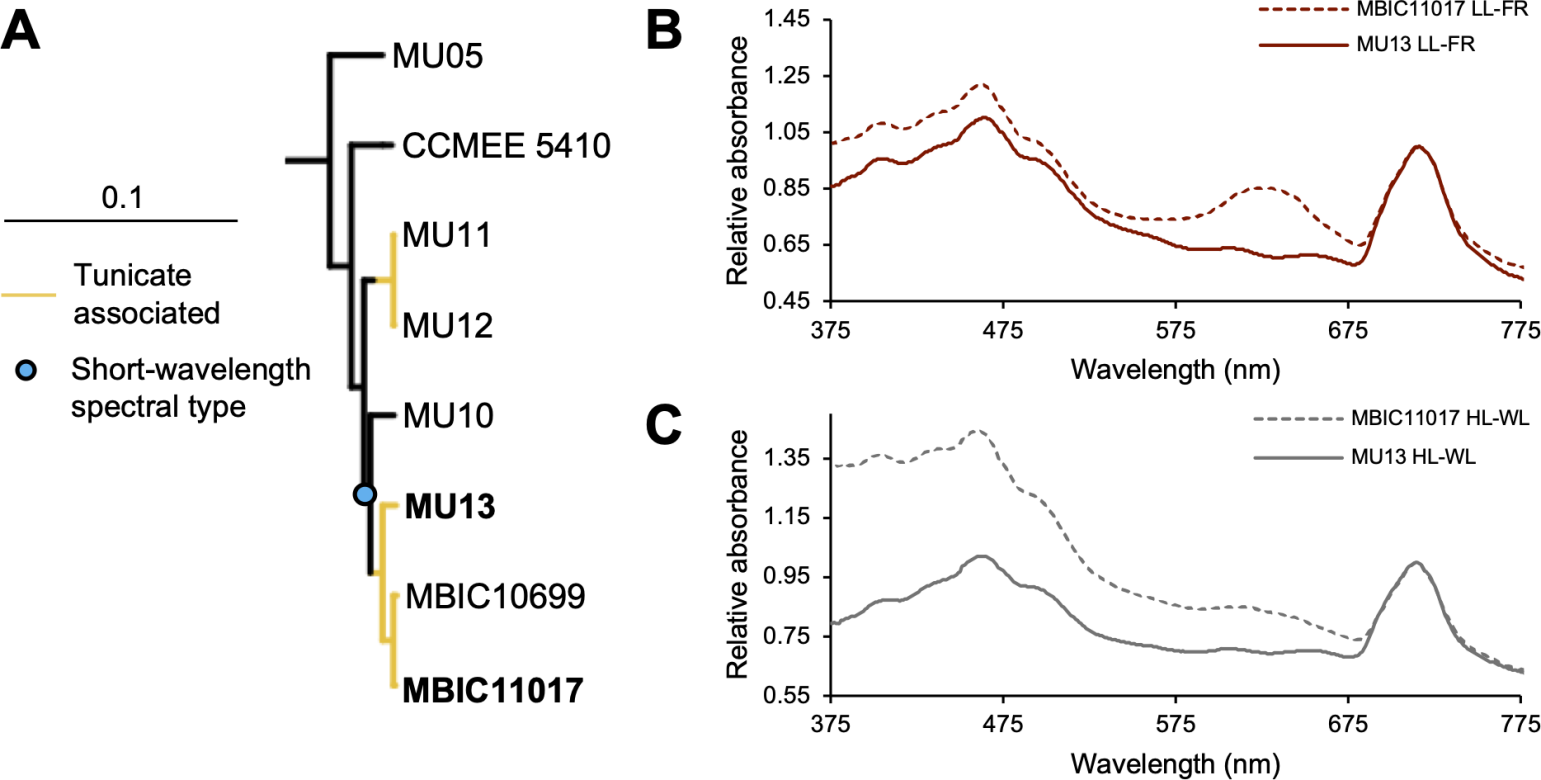
(A) Maximum likelihood phylogeny reconstructed from a genome-wide concatenation of protein sequences from 1283 single-copy orthologous genes of a subset of *Acaryochloris marina* strains. Branch lengths are in units of the expected number of amino acid substitutions per site. Bootstrap support was 100% for each node. Strains isolated from tunicates are signified by yellow branches and the blue dot indicates the node at which the short wavelength spectral type likely evolved (43). Absorption spectra of strains MBIC11017 and MU13 (bolded in A) from LL-FR (B) and HL-WL (C), normalized by the Chl *d* peak (713 nm). Note the prominent PC peak at 625 nm for MBIC11017 in LL-FR light.

PC-producing *A. marina* strain MBIC11017 and closely related strain MU13 (Fig. 1A; 98.3% genome-wide average nucleotide identity) were isolated from tropical tunicates (24), exhibit a blue-shifted light-harvesting phenotype compared with other *A. marina* (Fig. 1A) (43) and possess orthologous plasmids (pREB3 in MBIC11017) on which PC-related genes are located in MBIC11017 (13). These PC-related genes were lost in the *A. marina* ancestor and regained by horizontal transfer in MBIC11017 (13). The recently sequenced MBIC10699 is the closest relative of MBIC11017 among the *Acaryochloris* species reported so far (Fig. 1A), but it lacks this plasmid (44). This offers alternative possibilities regarding the timing of the origin of PC in *A. marina*: (i) PC genes were acquired by the common ancestor of MBIC10699 and MBIC11017, with the plasmid subsequently lost by the former strain; or (ii) PC genes were acquired via HGT after the split between MBIC10699 and MBIC11017 (44). Based on a divergence time of ~46 million years between *A. marina* strains MBIC11017 and CCMEE 5410 estimated by a Bayesian relaxed clock analysis (42), we conclude that PC genes were likely re-acquired by a strain MBIC11017 ancestor within the last 5 million years.

We investigated protein-coding gene expression differences between WL (30–35 μmol photons m^−2^ s^−1^) and FR (1–2 μmol photons m^−2^ s^−1^) environments (Fig. S1) for growing cells of MBIC11017 and MU13. MBIC11017 produced more PC in LL-FR than in HL-WL, whereas MU13 pigmentation was similar between environments (Fig. 1B). By contrast, Hernandez-Prieto et al. observed different light-harvesting strategies in MBICI11017 under high FR (20 μM m^−2^ s^−1^) and low WL (20 μM m^−2^ s^−1^) conditions, in which PC and most genes encoding subunits of photosystems showed high accumulation of transcripts in WL compared to FR (21). Therefore, if gene regulation is impacted predominantly by light intensity, we predicted that LL-FR conditions would induce PC and general light-harvesting machinery gene expression. Whereas in HL-WL, we predicted that PC and other photosynthesis genes would be downregulated, and genes associated with light stress, such as high light-inducible proteins (HLIPs), would exhibit higher expression. Because MU13 lacks PC, we predicted that the light response would involve fewer phenotypic changes between LL-FR and HL-WL environments.

**Fig 2:**
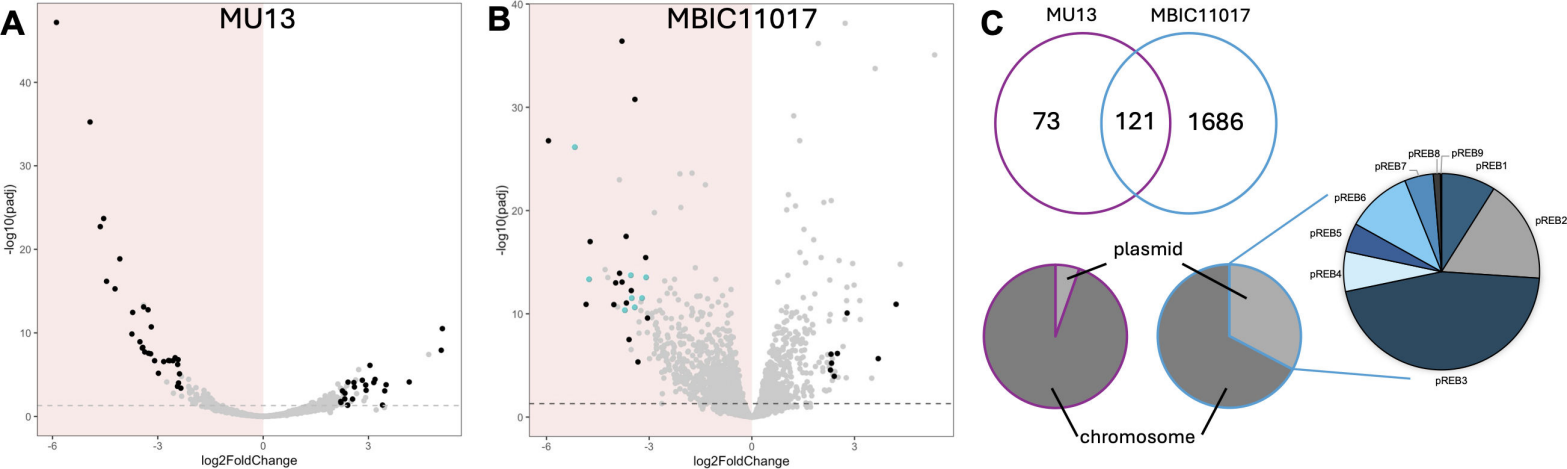
Genes expressed at higher rates in LL-FR (red background) and HL-WL (white background) environments in MU13 (A) and MBIC11017 (B). Most highly expressed genes that share the same expression patterns are in black. MBIC11017 PC genes are colored in cyan. (C) Venn diagram of the number and overlap of differentially expressed genes in LL-FR versus HL-WL for MU13 (pink) and MBIC11017 (blue). Pie charts display the proportion of DE genes that hit to chromosomes and plasmids and the proportion of DE genes on MBIC11017 plasmids (pREB1–9).

Of the 203 genes differentially expressed (DE) genes in MU13, 113 genes increased and 90 decreased expression with the shift to HL-WL (Fig. 2A). By contrast, a change in the light environment was associated with a massive remodeling of MBIC11017 gene expression (~500% more DE genes compared with MU13). In total, MBIC11017 contained 1806 DE genes in HL-WL, of which 704 genes increased and 1,102 genes decreased expression (Fig. 2B). PC genes were among the most highly decreased in HL-WL (Fig. 2B). One hundred twenty-one (60 %) of the DE genes in MU13 also exhibited differences in MBIC11017 expression (Fig. 2C).

A major difference between MBIC11017 and MU13 was the contribution of DE plasmid genes. Only 11 (5.4%) of DE genes in MU13 had top hits to plasmids using local BLAST queries (Fig. 2C). In contrast, nearly 33% of DE genes in MBIC11017 were located on one of its nine plasmids (Fig. 2C). Since plasmids make up ~20% of the MBIC11017 genome, this constitutes an enrichment of plasmid-encoded genes in the DE set. In particular, plasmids pREB2, pREB3, and pREB6 contributed large percentages of plasmid DE genes (17%, 46%, and 11%, respectively; Fig. 2). Despite being a smaller plasmid compared with some of the others, pREB6 made a significant contribution with 34% of its genes differentially expressed.

Differences in cellular copy number of pREB3 relative to the chromosome likely contributed to the DE patterns between HL-WL and LL-FR in MBIC11017: DNA sequence coverage indicated ~4 pREB3 copies per chromosome in LL-FR cells versus three copies in HL-WL (Fig. S1; other plasmids maintained similar stoichiometry between experimental conditions). Consequently, we would expect generally higher relative pREB3 expression in LL-FR, which was the case for 267 of the 270 DE pREB3 genes. The few pREB3 genes that exhibited higher expression in HL-WL included the PBS degradation gene *nblA* (as expected under PC-degradation conditions) and a two-component transcriptional regulator gene. Because this divergence in pREB3 copy number occurred during the 12 days of incubation under the experimental conditions following inoculation of the ancestral founder cell population, this result emphasizes that changes in gene copy stoichiometry over ecological time scales can impact gene expression (45).

### PC regulation in MBIC11017 is part of a conserved ancestral response to high light

**Fig 3:**
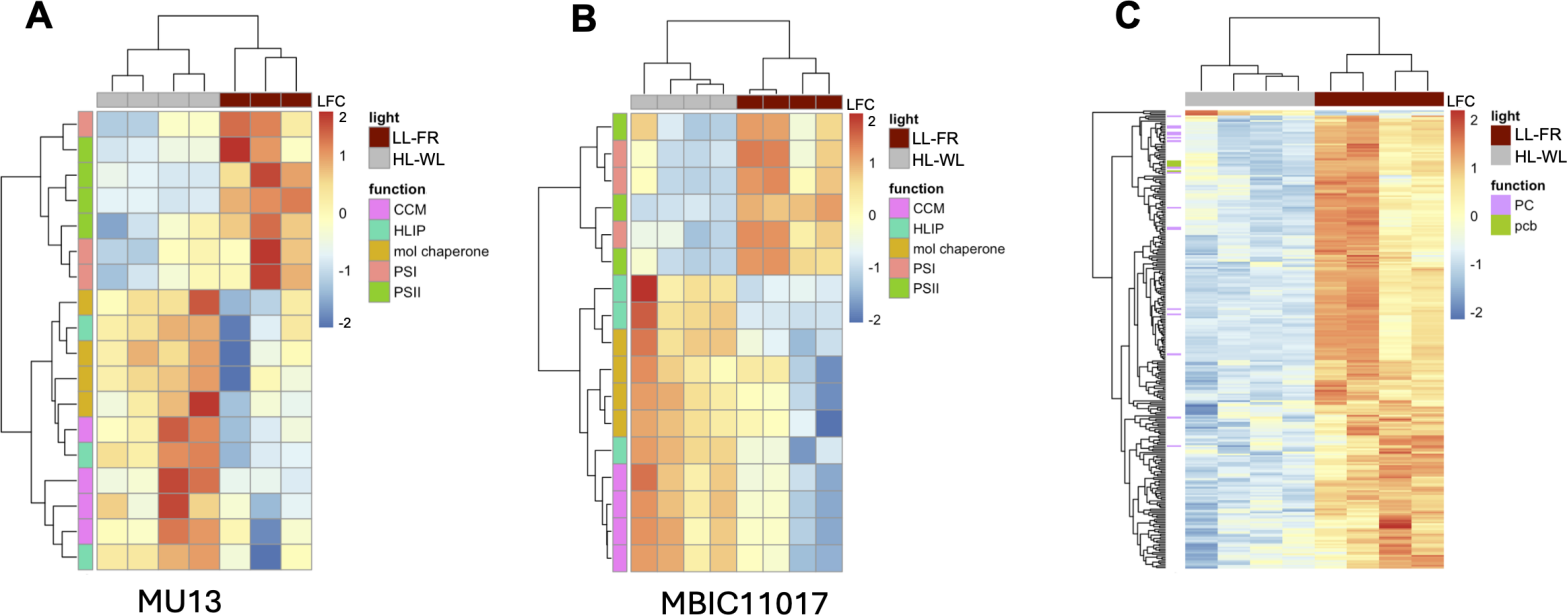
Heatmap of MU13 (A) and MBIC11017 (B) DE genes between LL-FR and HL-WL corresponding to carbon concentrating mechanism (CCM; pink), high light inducible proteins (HLIPs; blue), molecular chaperones (orange), PSI (coral), and PSII (green). (C) Heatmap of gene expression for MBIC11017 pREB3 plasmid genes (including all phycocyanin [PC) genes in purple) and select prochlorophyte Chl-binding protein genes (pcb; green) between LL-FR and HL-WL. Scales represent log_2_ fold change (LFC) in gene expression following DESeq2 normalization.

We observed hallmarks of the cyanobacterial high light response in MU13 under HL-WL, with increased expression of HLIPs, carbon dioxide concentrating mechanism (CCM) and molecular chaperone genes, along with downregulation of various photosynthesis genes (Fig. 3A; Tables 1 and 2). The CCM functions to elevate CO_2_ around the Rubisco active site (the enzyme involved in the first step of carbon fixation) to improve the overall efficiency of CO_2_ use (46, 47), and expression of CCM has been shown to be enhanced by HL conditions in model cyanobacteria such as *Synechocystis* sp. PCC 6803 (46, 48). HLIPs are localized in the thylakoid membrane and can serve a photoprotective function in HL by helping to dissipate excess absorbed light energy (49–51). Molecular chaperone-encoding genes (e.g., *groEL* and *dnaK*) are commonly induced by abiotic stresses, including a shift from LL to HL conditions (47). Pcb Chl-binding protein genes were constitutively expressed in both HL-WL and LL-FR but showed no differential expression. A subset (MBIC11017 orthologs: AM1_3655, AM1_1368, AM1_3654, AM1_1369, and AM1_1279) had significantly higher expression than the four other MU13 *pcb* copies. In LL-FR, PSI, and PSII genes (*psaD*, *psaF*, *psaJ, psbC*, *psbD*, *psbU*, *psbV*, and *psbY*) exhibited significantly increased expression (Fig. 3A; Table 2). These *psa* and *psb* genes encode the extrinsic proteins that are involved in electron transfer in PSI (52) and comprise, in part, the oxygen-evolving complex and enhance the structural stability of PSII (53), respectively.

**TABLE 1:** Top 35 most highly induced MU13 genes in HL-WL compared with LL-FR^*a*^

Gene ID	MBIC11017 ortholog	Annotation	Log_2_ (WL/FR)	*P* (adj)
**peg.1316**	**AM1_0314**	**Hypothetical protein**	**5.10688163**	**3.08E−11**
**peg.1315**	**AM1_0316**	**Hypothetical protein**	**5.07465423**	**1.20E−08**
peg.1314	AM1_0319	O-acetyl-ADP-ribose deacetylase	4.71863606	3.84E−08
**peg.5805**	**AM1_0325**	**ADP-ribosylglycohydrolase**	**4.16179969**	**7.55E−05**
**peg.7115**	**AM1_3292**	**Hypothetical protein**	**3.50230606**	**0.00016201**
**peg.1318**	**AM1_0288**	**HNH endonuclease family protein**	**3.4631609**	**0.00087167**
**peg.1465**	**AM1_5965**	**Nickel-binding accessory protein UreJ-HupE**	**3.40036271**	**0.0433439**
**peg.3292**	**AM1_4754**	**RNA-2′C3′-PO4:RNA-5′-OH ligase**	**3.18759739**	**3.67E−05**
**peg.1204**	**AM1_5383**	**Carbon dioxide concentrating mechanism protein CcmM**	**3.1517465**	**8.81E−05**
**peg.6573**	**AM1_3193**	**High light-inducible protein**	**3.04283813**	**7.66E−07**
**peg.6017**	**AM1_3712**	**Hypothetical protein**	**2.93385236**	**0.00075072**
**peg.2922**	**AM1_1454**	**Fasciclin domain protein**	**2.92158745**	**0.00017473**
peg.6874	No hit	Efflux ABC transporter permease protein	2.91903081	0.00544682
peg.6875	No hit	Efflux ABC transporter ATP-binding protein	2.8712256	0.01328938
**peg.1466**	**AM1_5964**	**Cobalamin biosynthesis protein CobW**	**2.82482345**	**4.66E−05**
peg.938	No hit	Hypothetical protein	2.72094096	2.49E−06
peg.811	AM1_4398	Conserved hypothetical protein	2.6623269	0.00186639
peg.4145	AM1_2331	ATP-dependent Clp protease proteolytic subunit ClpP (EC 3.4.21.92)	2.63568099	4.28E−05
**peg.4098**	**AM1_4447**	**ABC transporter ATP-binding protein**	**2.60043052**	**0.00030112**
**peg.6936**	**AM1_2753**	**Conserved hypothetical protein**	**2.58504642**	**8.81E−05**
**peg.1203**	**AM1_5382**	**Carbon dioxide concentrating mechanism protein CcmL**	**2.54804243**	**0.00836645**
peg.5462	AM1_1885	Hypothetical protein	2.50596909	0.01555429
**peg.1945**	**AM1_3636**	**Hypothetical protein**	**2.4177993**	**7.90E−05**
**peg.6753**	**AM1_2062**	**Hypothetical protein**	**2.40607749**	**0.04360515**
**peg.5806**	**AM1_0326**	**Hypothetical protein**	**2.40510067**	**0.04633216**
peg.4771	AM1_1506	Fructose-bisphosphate aldolase class II (EC 4.1.2.13)	2.38275191	0.00021242
**peg.4154**	**AM1_2322**	**Hypothetical protein**	**2.33021861**	**0.00166875**
**peg.4650**	**AM1_5322**	**Hypothetical protein**	**2.32581242**	**0.0080183**
peg.1949	AM1_3632	LSU ribosomal protein L21p	2.29949764	0.00058676
peg.4510	AM1_1095	Uncharacterized protein	2.29940318	0.01365635
peg.1296	AM1_4861	Tyrosine-protein kinase (EC 2.7.10.2)	2.28545439	0.00214841
**peg.2490**	**AM1_2644**	**Translation elongation factor G paralog VC2342**	**2.25904153**	**0.00088142**
peg.7086	AM1_2265	Hypothetical protein	2.23515782	0.04004659
**peg.3294**	**AM1_4752**	**Glutathione S-transferase (EC 2.5.1.18**)	**2.21492549**	**0.02170237**
**peg.6341**	**AM1_0722**	**Aspartyl/asparaginyl beta-hydroxylase**	**2.21124563**	**0.01631134**

^*a*^ Genes that share the same expression patterns as in MBIC11017 are bolded.

**TABLE 2:** Top 35 most highly induced MU13 genes in LL-FR compared with HL-WL^*a*^

Gene ID	MBIC11017 ortholog	Annotation	Log2 (WL/FR)	*P* (adj)
**peg.1855**	**AM1_5046**	**Photosystem II 12 kDa extrinsic protein (PsbU**)	**−5.887987827**	**6.74E−48**
**peg.1334**	**AM1_0272**	**Hypothetical protein**	**−4.930368867**	**5.77E−36**
**peg.1335**	**AM1_0271**	**Phage tail sheath protein FI**	**−4.639823424**	**1.93E−23**
**peg.1856**	**AM1_5047**	**Peptidyl-prolyl cis-trans isomerase**	**−4.543018287**	**2.07E−24**
**peg.1514**	**AM1_5907**	**Hypothetical protein**	**−4.460451197**	**6.72E−17**
**peg.924**	**AM1_1357**	**General secretion pathway protein G**	**−4.219338035**	**5.40E−16**
**peg.6297**	**AM1_0882**	**Pentapeptide repeat protein**	**−4.082122126**	**1.38E−19**
**peg.1336**	**AM1_0270**	**Hypothetical protein**	**−3.738013856**	**1.36E−10**
**peg.664**	**AM1_6045**	**Photosystem II protein D2 (PsbD**)	**−3.716582191**	**3.57E−13**
**peg.2106**	**AM1_3484**	**Conserved hypothetical protein**	**−3.509800007**	**1.16E−09**
**peg.5096**	**AM1_3686**	**Acetyltransferase putative**	**−3.442176162**	**6.56E−09**
**peg.1337**	**AM1_0269**	**Hypothetical protein**	**−3.427877887**	**5.43E−09**
**peg.1333**	**AM1_0273**	**Hypothetical protein**	**−3.405555453**	**7.86E−14**
peg.3036	no hit	Hypothetical protein	−3.404191773	4.71E−14
**peg.450**	**AM1_6406**	**Phage baseplate assembly protein**	**−3.372972634**	**1.94E−08**
**peg.4448**	**AM1_0601**	**Elongation factor G-like protein**	**−3.278557524**	**1.73E−13**
**peg.6296**	**AM1_0881**	**Hypothetical protein**	**−3.263160835**	**2.79E−08**
**peg.1332**	**AM1_0274**	**Hypothetical protein**	**−3.205752298**	**3.17E−08**
**peg.5093**	**AM1_3689**	**Phage tail collar**	**−3.186448361**	**1.91E−11**
**peg.1328**	**AM1_0278**	**Hypothetical protein**	**−3.091671369**	**2.14E−07**
**peg.2112**	**AM1_3478**	**Hypothetical protein**	**−2.983398656**	**6.85E−06**
**peg.5095**	**AM1_3687**	**Conserved hypothetical protein**	**−2.832052369**	**2.71E−07**
peg.253	AM1_6348	WD-repeat protein	−2.746448066	7.55E−05
**peg.1339**	**AM1_0267**	**Two-component transcriptional response regulator LuxR family**	**−2.694369203**	**2.02E−07**
**peg.1346**	**AM1_0258**	**Translation initiation factor 2**	**−2.667366298**	**2.14E−07**
**peg.5094**	**AM1_3688**	**Phage tail collar**	**−2.56959559**	**2.14E−07**
peg.2552	no hit	Hypothetical protein	−2.556177679	0.00165681
**peg.1338**	**AM1_2240**	**Two-component system sensor histidine kinase**	**−2.507389787**	**9.34E−08**
**peg.453**	**AM1_6403**	**Hypothetical protein**	**−2.440335449**	**0.00025142**
**peg.2107**	**AM1_3483**	**Hypothetical protein**	**−2.43483078**	**5.99E−07**
**peg.4968**	**AM1_1646**	**Universal stress protein**	**−2.423391336**	**1.58E−07**
**peg.3636**	**AM1_5144**	**Photosystem I subunit II (PsaD**)	**−2.410365962**	**9.91E−05**
**peg.2105**	**AM1_3485**	**Hypothetical protein**	**−2.383162876**	**7.96E−06**
**peg.4097**	**AM1_4448**	**Methyl-accepting chemotaxis protein**	**−2.343950018**	**0.00041142**

^*a*^ Genes that share the same expression patterns as in MBIC11017 are bolded.

While we observed a massive remodeling in MBIC11017 gene expression between LL-FR and HL-WL (Fig. 2B; Fig. S2), light-regulated gene modules were largely shared between MBIC11017 and MU13 (Fig. 3A and B), which indicates a conserved light response. In MBIC11017, CCM and HLIP genes had increased expression in HL-WL, whereas both extrinsic and subunit photosystem genes were substantially downregulated. Additional HLIP, heat shock protein, and regulator genes (e.g., PGR5) involved in stress responses were highly expressed in HL-WL when compared with MU13 (Table 3). We also observed significantly higher expression of MBIC11017 *pcb* genes AM1_3655, AM1_1368, AM1_3654, AM1_1369, and AM1_1279 in LL-FR (Fig. 3C), suggesting a general increase in light-harvesting capacity in these conditions.

**TABLE 3:** Top 35 most highly induced MBIC11017 genes in HL-WL compared with LL-FR^*a*^

Gene ID	Annotation	Location	Log_2_ (WL/FR)	*P* (adj)
AM1_1564	Hypothetical protein	Chr	5.34269819	8.37E−36
AM1_A0071	RNA-binding protein	pREB1	4.33899839	1.62E−15
**AM1_3193**	**High light-inducible protein**	**Chr**	**4.21758278**	**1.20E−11**
**AM1_3292**	**Hypothetical protein**	**Chr**	**3.69133468**	**2.20E−06**
AM1_1567	Hypothetical protein	Chr	3.60097025	1.74E−34
AM1_2559	Conserved hypothetical protein	Chr	3.50079909	0.00017223
AM1_2793	Hypothetical protein	Chr	3.16738048	3.59E−10
AM1_4584	High light-inducible protein	Chr	3.15312752	5.33E−12
AM1_2215	Hypothetical protein	Chr	2.98691072	1.68E−13
AM1_B0159	Hypothetical protein	pREB2	2.94515779	1.37E−15
**AM1_3636**	**Hypothetical protein**	**Chr**	**2.78305156**	**8.49E−11**
AM1_4488	Von Willebrand factor type A domain protein putative	Chr	2.77803963	2.70E−13
AM1_B0161	Conserved hypothetical protein	pREB2	2.76867776	5.39E−12
AM1_4588	PGR5 protein involved in cyclic electron flow	Chr	2.75755133	0.00015874
AM1_B0160	Hypothetical protein	pREB2	2.72569365	7.29E−39
AM1_3546	Conserved hypothetical protein	Chr	2.72009775	3.48E−10
AM1_4164	DUF897 domain membrane protein	Chr	2.66391969	1.47E−07
AM1_F0048	Ribonucleoside-diphosphate reductase beta subunit	pREB6	2.54832857	7.10E−16
AM1_5732	Hypothetical protein	Chr	2.51879697	0.00013869
**AM1_5382**	**Carbon dioxide concentrating mechanism protein CcmL**	**Chr**	**2.4992051**	**7.01E−07**
AM1_B0158	Hypothetical protein	pREB2	2.42699604	3.94E−05
**AM1_5380**	**Carbon dioxide concentrating mechanism protein, putative**	**Chr**	**2.40738354**	**0.00011214**
AM1_E0044	Hypothetical protein	pREB5	2.36721304	8.17E−05
AM1_1214	Hypothetical protein	Chr	2.35268273	0.00028175
AM1_4165	Conserved hypothetical protein	Chr	2.35174018	2.22E−06
**AM1_5381**	**Carbon dioxide concentrating mechanism protein CcmK**	**Chr**	**2.32867562**	**6.03E−06**
AM1_B0409	Conserved hypothetical protein	pREB2	2.31996685	1.09E−21
**AM1_2753**	**Conserved hypothetical protein**	**Chr**	**2.31344772**	**8.21E−07**
**AM1_5383**	**Carbon dioxide concentrating mechanism protein CcmM**	**Chr**	**2.2970618**	**2.81E−05**
AM1_F0047	Ribonucleotide-diphosphate reductase alpha subunit	pREB6	2.28937714	1.17E−10
AM1_4610	Hypothetical protein	Chr	2.2785254	5.46E−14
AM1_3819	Hypothetical protein	Chr	2.27334948	1.24E−05
AM1_2642	Hypothetical protein	Chr	2.26035697	1.51E−05
AM1_5670	Hypothetical protein	Chr	2.25934153	0.00232397
AM1_1464	Heat shock protein (HSP20) family protein	Chr	2.25927709	5.05E−10

^*a*^ Genes that share the same expression patterns as in MU13 are bolded.

Many pREB3 genes are PC-associated, and expression patterns of these PC genes followed the expected HL/LL response (Fig. 2B; Fig. 3C). In HL, genes involved in phycobiliprotein assembly are usually downregulated in order to reduce antenna size and thus minimize absorption of excess light energy (54). As expected, we observed significantly lower expression of PC genes in HL-WL compared with LL-FR (Fig. 3C; Table 4). For example, *cpcB* genes were among the most downregulated genes in HL-WL with a log_2_ fold change (LFC) ranging from −5.2 to −3.5 (*P* < 4.56 × 10^−11^). PBS linker (*cpcCD*) and rod-core linker (*cpcL*) genes also followed the same expression patterns as *cpcB* (Fig. 3C; Table 4). Expression of *nblA* increased in HL-WL compared to LL-FR, following the typical HL response. We also observed DE regulatory genes potentially involved in MBIC11017 PC regulation. For example, a pREB3 gene AM1_C0036 that encodes an RNA polymerase sigma factor showed significantly higher expression in conditions where PC is induced. In fact, it was the most highly expressed sigma factor in the transcriptome in LL-FR (LFC: −3.923, *P* = 2.14 × 10^−11^; Table 4). We conclude that PC assembly and degradation have been re-assimilated into a conserved ancestral HL/LL response.

**TABLE 4:** Top 35 most highly induced MBIC11017 genes in LL-FR compared with HL-WL^*a*^

Gene ID	Annotation	Location	Log_2_ (WL/FR)	*P* (adj)
**AM1_5046**	**Photosystem II 12 kDa extrinsic protein PsbU**	**Chr**	**−5.942704**	**1.72E−27**
AM1_C0098	Phycocyanin beta subunit	pREB3	−5.1709918	7.11E−27
**AM1_0882**	**Pentapeptide repeat protein**	**Chr**	**−4.8421117**	**1.23E−11**
AM1_C0192	Phycocyanin beta subunit	pREB3	−4.7540007	4.59E−14
AM1_G0114	Photosystem II 12 kDa extrinsic protein PsbU	pREB7	−4.7239533	1.01E−17
AM1_C0039	Hypothetical protein	pREB3	−4.2871428	5.30E−15
AM1_C0038	Hypothetical protein	pREB3	−4.2078844	3.12E−14
**AM1_1646**	**Universal stress protein**	**Chr**	**−4.0316068**	**1.29E−11**
**AM1_3688**	**Phage tail collar**	**Chr**	**−3.9781575**	**1.02E−13**
AM1_C0036	RNA polymerase sigma-2 subunit domain protein	pREB3	−3.9224959	2.14E−11
AM1_3684	Conserved hypothetical protein	Chr	−3.8700611	1.06E−23
**AM1_3689**	**Phage tail collar**	**Chr**	**−3.8652407**	**1.20E−14**
**AM1_1445**	**Light-independent protochlorophyllide reductase subunit L**	**Chr**	**−3.7952815**	**3.94E−37**
**AM1_3687**	**Conserved hypothetical protein**	**Chr**	**−3.7924153**	**8.56E−14**
AM1_C0100	Phycocyanin beta subunit	pREB3	−3.7092764	4.56E−11
**AM1_3686**	**Acetyltransferase putative**	**Chr**	**−3.6736317**	**3.17E−18**
**AM1_5047**	**Peptidyl-prolyl cis-trans isomerase**	**Chr**	**−3.6654404**	**9.00E−12**
**AM1_3119**	**Conserved hypothetical protein**	**Chr**	**−3.5883042**	**3.10E−08**
AM1_C0212	Phycocyanin beta subunit	pREB3	−3.5354092	1.89E−14
AM1_1114	Conserved hypothetical protein	Chr	−3.5294168	2.94E−07
**AM1_5259**	**Hypothetical protein**	**Chr**	**−3.5228114**	**5.69E−13**
AM1_C0094	Phycobilisome 32.1 kDa linker polypeptide	pREB3	−3.5085456	3.04E−12
AM1_C0203	Phycobilisome rod-core linker polypeptide CpcG	pREB3	−3.4219357	2.40E−11
**AM1_1444**	**Light-independent protochlorophyllide reductase N subunit**	**Chr**	**−3.4172067**	**1.71E−31**
**AM1_3483**	**Hypothetical protein**	**Chr**	**−3.3282291**	**4.60E−06**
AM1_C0034	Hypothetical protein	pREB3	−3.2968688	7.55E−09
AM1_C0030	Conserved hypothetical protein	pREB3	−3.294875	6.05E−10
AM1_C0214	Hypothetical protein	pREB3	−3.2429328	4.89E−11
AM1_3690	Phage tail collar	Chr	−3.2406139	7.10E−12
AM1_C0216	Phycobilisome linker protein	pREB3	−3.2073353	3.04E−12
**AM1_1539**	**Light-independent protochlorophyllide reductase B subunit**	**Chr**	**−3.1015886**	**3.61E−16**
AM1_C0093	Phycobilisome linker protein	pREB3	−3.0893309	3.00E−14
AM1_2019	Conserved TM helix repeat-containing protein	Chr	−3.0540738	6.34E−09
**AM1_3509**	**Aquaporin Z**	**Chr**	**−3.0483277**	**2.60E−10**
AM1_C0035	Hypothetical protein	pREB3	−3.0480564	8.90E−11

^*a*^ Genes that share the same expression patterns as in MU13 are bolded; gray-shaded are phycocyanin (*Cpc*) genes.

The gene expression patterns we observed (Fig. 3) with respect to light response between FR and WL are opposite to Hernandez-Prieto et al. (21), which examined MBIC11017 expression differences in lower WL (20 μmol m^−2^ s^−1^) and higher FR (20 μmol m^−2^ s^−1^) light environments. However, the gene expression patterns we observed in PC-inducing conditions are consistent across both studies. This indicates that PC regulation in MBIC11017 is primarily a response to light intensity (HL/LL). Contrary to Hernandez-Prieto et al. (21), we observed higher expression of *pcb* genes in PC-inducing conditions, suggesting that PC and Pcbs both have roles in light harvesting at low light intensities by increasing antenna size (55). We observed constitutively high expression of MU13 *pcb* orthologs, indicating that the same *pcb* genes are important to light harvesting across both strains, but they exhibit greater light-dependent plasticity in MBIC11017.

### Potential mechanisms of PC gene regulatory assimilation

In cyanobacteria, the regulation of the high light response is mediated by the highly conserved two-component system comprising sensor histidine kinase NblS and response regulator RpaB (29, 34, 36). In two-component systems, the sensor histidine kinase senses external stimuli and changes the phosphorylation state of the response regulator, which subsequently alters expression of target genes (29, 56). NblS is involved in a number of processes critical for altering the photosynthetic apparatus in response to HL (29, 38, 57); an *nblS* mutant of *Synechococcus elongatus* PCC 7942 is unable to properly control photosynthesis-related genes (i.e., *psa* and *cpcBA*) in HL (38, 41). RpaB is an important cyanobacterial response regulator that is involved in both light acclimation responses and circadian clock-related processes (58). RpaB can act as either an activator or repressor of transcription. RpaB binds to HLR1 (high light regulatory 1) element (s) ~50–100 nucleotides upstream of transcription start sites to activate photosynthesis genes (e.g., PC-associated genes) in LL (40, 59, 60). By contrast, in LL, RpaB negatively regulates HLIP and *nblA* genes by binding to HLR1 elements near the transcription start site to repress both PBS degradation and HLIP gene expression (61); whereas, in HL, RpaB becomes unbound, mediated by NblS, resulting in a de-repression of both HLIPs and *nblA* for photoprotection and PBS disassembly, respectively (40, 61).

**Fig 4:**
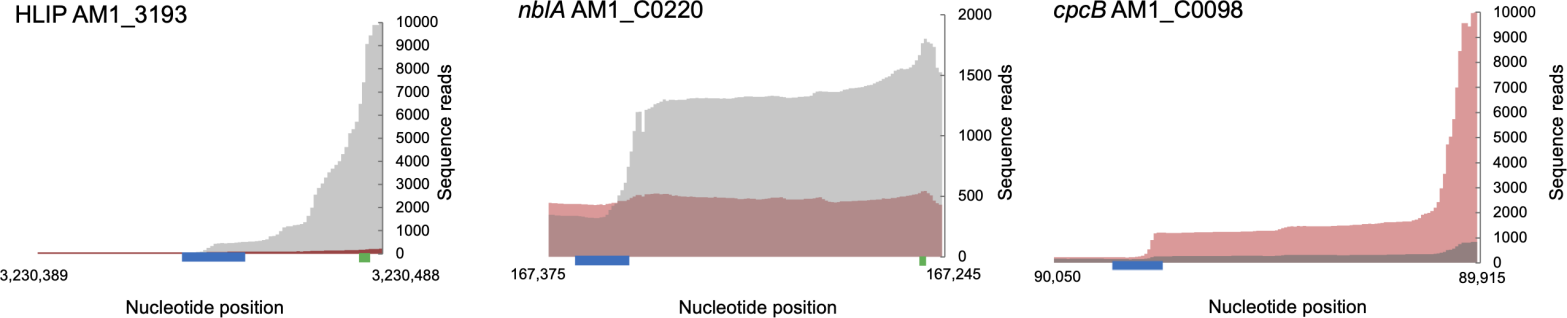
Location of putative HRL1 elements (blue boxes; *P* < 10^−4^). HLIP AM1_3193 and *nblA* AM1_C0220 show examples of high light induction, where RpaB is a putative repressor of transcription in low light by interfering with the promoter/RNA polymerase. *cpcB* AM1_C0098 is an example of RpaB as a putative activator in low light with the binding site 50–100 nucleotides upstream. *nblA* and *cpcB* are plotted in reverse orientation. Red shading corresponds to mapped reads at LL-FR and gray is mapped reads at HL-WL. Green boxes are start codon locations (where applicable; start for *cpcB* is farther downstream of window shown).

Given that the PC-associated genes appear to have reassimilated into a conserved ancestral light response, we expected to find potential signatures of HLR1 regulatory elements associated with the NblS-RpaB two-component system. We searched for putative HLR1 elements in the MBIC11017 genome using the *Synechocystis* PCC 6803 consensus sequence motif (G/T)TTACA(T/A)(T/A)NN(G/T)TTACA(T/A)(T/A). On the MBIC11017 chromosome, motif searches identified potential HLR1 elements near the putative transcription start sites of HLIP genes in our gene expression data, as expected for repression in LL (Fig. 4). We also found strong HLR1 element candidates near PC-related genes on pREB3. For example, a putative HLR1 element binding site is near the transcription start site of *nblA*, and we observed a dramatic increase in expression during WL compared to FR, suggesting a bound RpaB (or homologous response regulator) could potentially act as a repressor during LL-FR (Fig. 4). A putative HLR1 element was much farther (~100 nucleotides) from the putative transcription start site of *cpcB*, potentially enabling RpaB to bind and activate transcription in LL-FR (Fig. 4). These results suggest that RpaB (or homologous protein) contributes to the regulation of PC expression patterns. There are seven total *rpaB* homologs in MBIC11017, two of which are located on pREB3. One of these (AM1_C0101) is a promising candidate for PC regulation, because it was strongly induced in FR with PC-related genes. Taken together, we propose that horizontally acquired PC genes likely brought their regulatory elements with them, which could be recognized by *A. marina*’s pre-existing light response machinery.

Ultimately, this study provides an example of how the violation of Dollo’s Law (i.e., the proposition that the loss of a complex trait is irreversible) is possible (13, 62, 63). Because of their pleiotropic roles in multiple physiological stress responses, important regulators of PBS production and disassembly including NblS and RpaB were functionally retained after PBS genes were lost in the *Acaryochloris* ancestor. When PC genes were reacquired in MBIC11017, they likely brought along their regulatory elements (e.g., HLR1 elements) that could reactivate a proper regulatory response, even after millions of years of *Acaryochloris* diversification. This was likely facilitated by the location of all PC-acquired genes on one plasmid and the common capacity of bacteria to organize functionally related genes as operons. Future work seeks to characterize the identity of the regulator(s) that bind to these putative HLR1 elements in *A. marina*.

## MATERIALS AND METHODS

### Phylogenetics

A genome-wide phylogeny was reconstructed for *A. marina* strains (NCBI BioProject PRJNA649288), including the recently described MBIC10699 (44) (NCBI BioProject PRJDB13468). A total of 1,283 single copy groups of orthologous coding sequences were identified by OrthoFinder v2.2.7 (64, 65), which then inferred a multiple sequence alignment single copy ortholog protein sequences of using MAFFT v7 (66). We constructed a maximum likelihood tree with 1,000 ultrafast bootstrap replicates (67) using IQ-TREE version 2.0 (68) according to the JTT + F + R10 model of sequence evolution selected by AIC in ModelFinder (69). A subset of this tree was included in this paper.

### Gene expression experiment

Cells of *A. marina* strains MU13 and MBIC11017 were grown in 150 mL of modified IO-BG11 media [as in reference (13)] in 250 mL flasks. Four independent cultures of each strain were propagated into 30–35 µmol photons m^−2^ s^−1^ cool white fluorescent light (HL-WL) or 1.5–2 µmol photons m^−2^ s^−1^ LED light (LL-FR; 710 nm emission peak) in 12-h light:dark cycles at 30°C. See Fig. S3 for spectra of each light environment. Growth was measured every 48 h by taking OD_750_ readings of a 2-mL subsample using a Beckman Coulter DU 530 spectrophotometer (Indianapolis, IN), after which cultures were randomly moved to different positions within each respective light environment to mitigate any differences in light exposure. To characterize *in vivo* pigment absorbance, wavelength scans (300–800 nm) of cultures were measured using a Beckman Coulter DU 530 spectrophotometer (Indianapolis, IN). During the light period, mid-exponentially growing cells (OD_750_ ~0.2) were filtered, immediately frozen in liquid nitrogen, and stored at −80°C until extraction. RNA was extracted using the Omega EZNA Plant RNA extraction kit, and libraries were prepared using Zymo-Seq RiboFree Total RNA Library Kit. cDNA libraries were then paired-end sequenced using the Illumina Novaseq 6000 S4 system. RNA sequence data can be found at NCBI BioProject PRJNA1130970.

Raw sequence reads were trimmed with trimmomatic v0.36 (70) using default settings to remove any low-quality reads and adaptor sequences. Paired reads were then combined when possible using flash 1.2.11 (71). Sortmerna v4.3.4 (72) was used to remove any rRNA reads. The remaining reads were aligned to either the published MBIC11017 reference genome (73) or the MU13 draft genome assembly (24) using bowtie2 v2.3.4.3 (74). Reads were counted for CDS, tRNA, and pseudogene features using htseq-count v2.0.2 (75). Final read counts were then analyzed to identify differentially expressed genes using DESeq2 (76) in R v4.2.1. Obtained *P* values were adjusted using the Benjamini and Hochberg method (*P* adj). We used eggNOG to assign Clusters of Orthologous Genes categories and KO (Kyoto Encyclopedia of Genes and Genomes [KEGG] Orthology) IDs to annotated MU13 and MBIC11017 DE genes with default settings (77, 78) and included these in supplemental information (Tables S1 and S2).

### Coverage estimation of genome and plasmids

DNA was extracted from frozen pellets using the DNeasy PowerBiofilm DNA extraction kit (Qiagen) following manufacturer instructions. Sample libraries were prepared and sequenced on an Illumina NextSeq2000 platform (150 bp paired-end) at SeqCoast Genomics (Portsmouth, NH). We used breseq v0.38.3 (79) to identify the coverage of the chromosome and plasmids between MBIC11017 HL-WL and MBIC11017 LL-FR raw sequence reads. Genome sequence data can be found at NCBI BioProject # PRJNA1130970.

### Identification of putative HLR1 elements

To identify candidate HLR1 elements in *A. marina* genomes, we used both FIMO (80) and MCAST (81), available as part of the MEME Suite software toolkit (https://meme-suite.org). Specifically, we searched for matches to the motif (G/T)TTACA(T/A)(T/A)NN(G/T)TTACA(T/A)(T/A), which is the HLR1 element consensus sequence in *Synechocystis* PCC 6803 (82).

## Data Availability

The data sets analyzed during the current study are deposited at NCBI under BioProject PRJNA649288 and PRJDB13468. RNAseq reads generated during this study are deposited in the NCBI SRA repository under BioProject PRJNA1130970.

## SUPPLEMENTAL MATERIAL

Supplemental Table Captions mbio.02423-24-s0001.docx Table S1 and S2 captions.

Supplemental Figures mbio.02423-24-s0002.docx Figures S1-S3.

Table S1 mbio.02423-24-s0003.xlsx All differentially expressed genes in MU13 between LL-FR and HL-WL environments.

Table S2 mbio.02423-24-s0004.xlsx All differentially expressed genes in MBIC11017 between LL-FR and HL-WL environments.

ASM does not own the copyrights to Supplemental Material that may be linked to, or accessed through, an article. The authors have granted ASM a non-exclusive, world-wide license to publish the Supplemental Material files. Please contact the corresponding author directly for reuse.
