# Supplementary material for: Integration of horizontally acquired light-harvesting genes into an ancestral regulatory network in the cyanobacterium Acaryochloris marina MBIC11017
Source: mBio. 2024 Nov 18;15(12):e02423-24. doi: 10.1128/mbio.02423-24 (PMC11633204; doi:10.1128/mbio.02423-24)
Supplement: Supplemental Table Captions — Table S1 and S2 captions. [file mbio.02423-24-s0001.docx]

**Table S1.** All differentially expressed genes in MU13 between LL-FR and HL-WL environments. Clusters of Orthologous Genes categories and KO (Kyoto Encyclopedia of Genes and Genomes [KEGG] Orthology) IDs are included.

SI_MU13_DE_GO.xlxs

**Table S2.** All differentially expressed genes in MBIC11017 between LL-FR and HL-WL environments. Clusters of Orthologous Genes categories and KO (Kyoto Encyclopedia of Genes and Genomes [KEGG] Orthology) IDs are included.

SI_MBIC11017_DE_GO.xlxs
