# Supplementary material for: Integration of horizontally acquired light-harvesting genes into an ancestral regulatory network in the cyanobacterium Acaryochloris marina MBIC11017
Source: mBio. 2024 Nov 18;15(12):e02423-24. doi: 10.1128/mbio.02423-24 (PMC11633204; doi:10.1128/mbio.02423-24)
Supplement: Supplemental Figures — Figures S1-S3. [file mbio.02423-24-s0002.docx]

**Figure S1**. Coverage distributions of MBIC11017 chromosome (black) and plasmids (gray, except for pREB3 in dark blue and pREB6 in light blue, corresponding to Fig. 2C) in HL-WL and LL-FR.


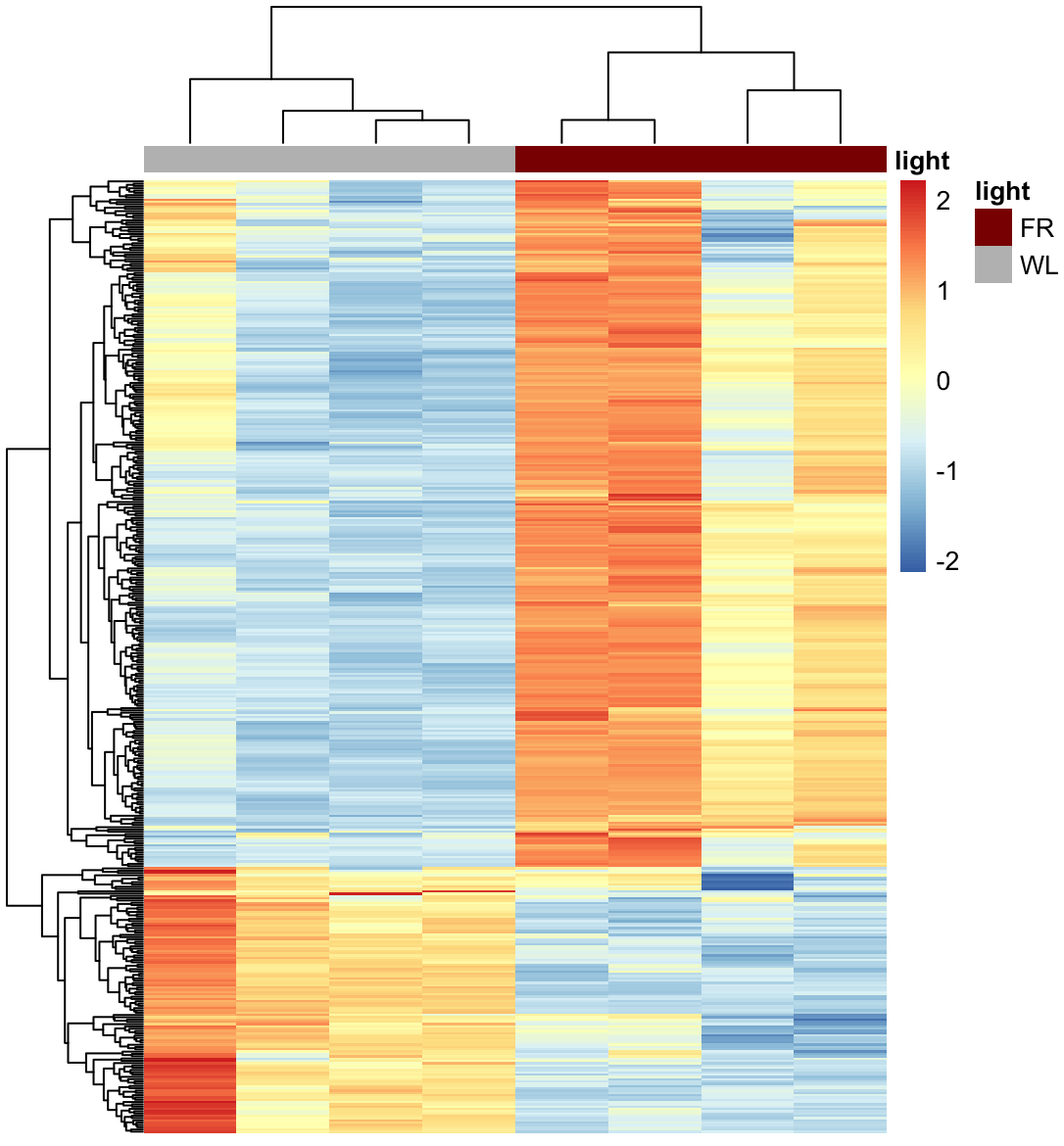


LL-FR

HL-WL

LFC

**Figure S2**. Heatmap of MBIC11017 full transcriptome of DE genes between HL-WL and LL-FR. Scales represent log_2_ fold change (LFC) in gene expression following DESeq2 normalization.

**Figure S3**. Spectra of cool white fluorescent light (WL) and far-red LED light (FR) environments.
